# Patients with chronic hepatitis C receiving sofosbuvir and ribavirin-based treatment, with or without interferon in Zhejiang, China: An observational study

**DOI:** 10.1097/MD.0000000000012403

**Published:** 2018-09-21

**Authors:** Xiao-Wei Xu, Xiao-Xin Wu, Ke-Da Chen, Da-Zhi Chen, Hui-Lin Ou, Jun-Wei Su, Hai-Ying Yu, Hang-Ping Yao, Lan-Juan Li

**Affiliations:** State Key Laboratory for Diagnosis and Treatment of Infectious Diseases, Collaborative Innovation Center for Diagnosis and Treatment of Infectious Diseases, The First Affiliated Hospital, School of Medicine, Zhejiang University, Hangzhou, Zhejiang, China.

**Keywords:** chronic hepatitis C, rapid virological response, ribavirin, sofosbuvir, sustained virological response

## Abstract

Hepatitis C virus (HCV) is one of the most important virus as the cause of liver disease in China. The aim of the present study was to explore whether sofosbuvir and ribavirin-based treatment can cure patients with chronic hepatitis C in eastern China. We examined a cohort of HCV-monoinfected patients and 9 patients agreed to participate in our treatment and research. The patients were diagnosed with chronic hepatitis C with or without cirrhosis. Nine patients including 4 female and 5 male met the requirements for selection and were willing to participate in this experiment. Sofosbuvir and ribavirin-based treatment with or without interferon was given to the patients. Viral loads, cytokines, and chemokines were recorded during treatment and after treatment. After 2 weeks of sofosbuvir and ribavirin-based treatment, the viral load of patients decreased to limits of detection. Eight patients were cured. Patients had rapid virological response (RVR) with undetectable viral load at week 4 and sustained virological response (SVR). The interferon-inducible protein-10 (IP-10) decreased after the treatment. However, the patient with cirrhosis failed, as the virus reappeared during SVR4. At the same time, the IP-10 dramatically increased as the relapse of the HCV virus. In summary, the IP-10 has the potential to be the biomarker for the prognostic of HCV.

## 1 Introduction

Hepatitis C virus (HCV) is one of the most important virus as the cause of liver disease. Globally, the number of infections was estimated more than 185 million. According to the World Health Organization (WHO), over 71 million people worldwide have chronic hepatitis C infection. Approximately 399,000 people die each year from hepatitis C, mostly of liver cirrhosis and hepatocellular carcinoma.^[1–4]^ In China, the HCV prevalence rate is about 0.43%. In the general population, the number of infections was estimated about 5.6 million.^[5–7]^ There is currently no effective preventive hepatitis C vaccine available. In a sense, the problem of HCV was more severe than hepatitis B.

The treatment of HCV was difficult and time-consuming. Before the availability of direct-acting antiviral (DAA) drugs, the combined use of pegylated-interferon (PEG-IFN) and ribavirin (RBV) was the standard of treatment for chronic HCV infection. To achieve a sustained virological response (SVR) was the goal of treatment. A SVR was defined as undetectable HCV viral load 12 or 24 weeks at the end of treatment. The previous treatment strategy was toxic and less effective, but the DAA drugs are totally different. The performance of new agents was reported in various researches.^[8,9]^

Sofosbuvir (SOF), a NS5B polymerase inhibitor, is one of potential DAA drugs and now under clinical trial in many countries, including the United States, France, and Canada.^[8–13]^ It has been reported pan-genotypic antiviral active with a high barrier to resistance. It has been proved to be effective for infections with genotypes 1–6 in numerous settings in combination with other antivirals.^[8]^ The success of SOF was very encouraging.

In order to make sure whether SOF and RBV-based treatment was effective for patients in Zhejiang, we conducted this study. The cytokines and chemokines expression is known to be interfered by HCV. Inflammatory cytokines or chemokines during SOF and RBV therapy for genotype 2 and 3 was reported before, but only 4 parameters were investigated.^[14]^ Another study explored the alterations in the systemic inflammatory cytokine and chemokine milieu in patients with chronic hepatitis C.^[15]^ Hence, we hypothesized that the level of serum cytokines and chemokines might be associated with the outcome of patients in response to anti-HCV treatment and has the potential of being the biomarker for the prognosis of HCV. To test this possibility, a total of 38 cytokines and chemokines were observed during the SOF and RBV-based therapy. Meanwhile, the viral loads were also recorded during treatment and after treatment.

## 2 Methods

### 2.1 Ethical approval

Patients with chronic hepatitis C were recruited between October 2015 and October 2016. Patients with human immunodeficiency virus or other hepatitis virus were excluded. The study was conducted with approval of the ethics committee of the First Affiliated Hospital, College of Medicine, Zhejiang University. The Ethical approval number is 2015–56. All patients involved in this study gave their informed consents. All samples were collected after informed written consents were obtained from all the participants.

### 2.2 Study design

This clinical trial is a prospective cohort study. Our hospital was one of the centers of this international, multicenter, open-label, stage 3b (NCT02021643) study. Serum samples were collected from 9 patients diagnosed with chronic HCV. The diagnosis of HCV infection depends on positive detection of HCV RNA by reverse transcription polymerase chain reaction. Nine patients including 4 female and 5 male met the requirements for selection and were willing to participate in this experiment. The cases positive for genotype 1 (n = 7), 2 (n = 1), and 6 (n = 1). Viral loads, cytokines, and chemokines were recorded during treatment and after treatment.

### 2.3 Biochemical and virological assessments

HCV RNA was analyzed by using the Roche HCV Test, version 2.0 for Use with the High Pure System (Roche Molecular Systems, Inc., Branchburg, NJ), which has a lower limit of quantification of 25 IU/mL. HCV genotyping was performed using the Siemens VERSANT HCV Genotype INNO-LiPA 2.0 Assay (Siemens Healthcare Diagnostics, Inc., West Sacramento, CA).

### 2.4 Cytokines and chemokines assay

Serum samples were collected by centrifugation of blood at 1000*g* for 10 minutes and stored immediately at -80°C degree until use. Expressions of 38 cytokines and chemokines, including expression of epidermal growth factor (EGF), fibroblast growth factors (FGF-2), transforming growth factor (TGF)-α, Eotaxin, granulocyte-macrophage colony stimulating factor (GM-CSF), Fractalkine, granulocyte colony stimulating factor (G-CSF), IFN (Interferon) α-2, IFNγ, growth-related oncogene (GRO), Fms-like tyrosine kinase receptor 3 ligand (Flt-3L), interleukin (IL)-10, monocyte chemotactic protein (MCP)-3, IL-12 (p40), macrophage-derived chemokine (MDC), IL-12 (p70), IL-13, IL-15, sCD40L, IL-17A, IL-1RA, IL-1α, IL-9, IL-1β, IL-2, IL-3, IL-4, IL-5, IL-6, IL-7, IL-8, MCP-1, macrophage inflammatory protein (MIP)-1α, MIP-1β, tumor necrosis factor (TNF)α, TNFβ, vascular endothelial growth factor (VEGF), interferon-inducible protein-10 (IP-10) were measured by MILLIPLEX MAP Human Cytokine/Chemokine-Premixed 38 Plex (EMD Millipore Corporation, Billerica, MA) using Luminex 200 instrument (EMD Millipore Corporation, Billerica, MA) according to the manufacturer instructions. All samples were analyzed in 1 run.

### 2.5 Statistical analysis

Statistical analysis of the results was performed using Graphpad prism 5 (GraphPad Software, Inc., La Jolla, CA). One-way analysis of variance (ANOVA) followed by Tukey test was used to analyze the results. *P* value < .05 was considered significant.

## 3 Results

### 3.1 The characteristics of patients and therapeutic outcome

Nine patients including 4 female and 5 male participated in this experiment. The mean age of patients is 47. Different treatment was given to each patient. The detailed clinical characteristics were as follows.

### 3.2 Case 1

Case 1 is a 44-year-old male with no significant medical history before being diagnosed with HCV genotype 1b in 2014 (Table 1). Before starting our treatment, his viral load was 10,400,000 IU/mL. The strategy SOF+RBV+PEG-IFN was given. The course of treatment was 12 weeks. He had a rapid virological response (RVR) with undetectable viral load at week 4 that remained undetectable for the remainder of 10 weeks of SOF-based triple therapy. The strategy SOF+RBV+PEG-IFN was stopped after the 12-week therapy. A sustained virologic response (SVR) was observed. The viral load remained undetectable until SVR24 (Table 2).

**Table 1:**
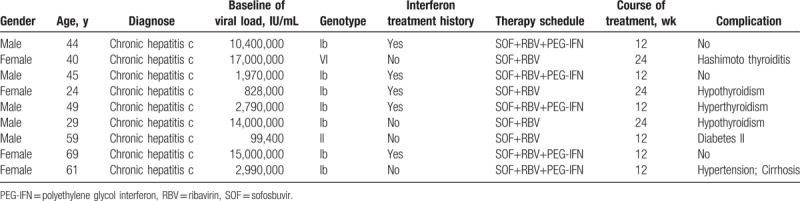
The basic information and treatment strategy of 9 patients.

**Table 2:**
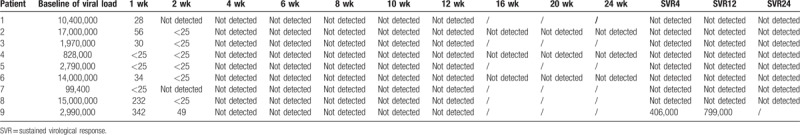
The viral load changing in nine patients during the process of treatment.

### 3.3 Case 2

Case 2 is a 40-year-old female with Hashimoto thyroiditis before being diagnosed with HCV genotype 6 in 2014 (Table 1). This patient was previously taken no treatment. Before starting our treatment, his viral load was 17,000,000 IU/mL. For the consideration of uncontrolled Hashimoto thyroiditis, PEG-IFN was not suitable for her. The strategy SOF+RBV was given for 24 weeks. She had a RVR with undetectable viral load at week 4 that remained undetectable for the remainder of 20 weeks of SOF+RBV therapy. The strategy SOF+RBV was stopped after the 24-week therapy. A SVR was observed. The viral load remained undetectable until SVR24 (Table 2).

### 3.4 Case3

Case3 is a 44-year-old male with no significant medical history before being diagnosed with HCV genotype 1b in 2007 (Table 1). Before starting our treatment, his viral load was 1,970,000 IU/mL. The strategy SOF+RBV+PEG-IFN was given. He had a RVR with undetectable viral load at week 4 that remained undetectable for the remainder of 10 weeks of SOF-based triple therapy. The strategy SOF+RBV+PEG-IFN was stopped after the 12-week therapy. A SVR was observed. The viral load remained undetectable until SVR24 (Table 2).

### 3.5 Case 4

Case 4 is a 24-year-old female with hypothyroidism before being diagnosed with HCV genotype 1b in 2014 (Table 1). Before starting our treatment, her viral load was 828,000 IU/mL. The strategy SOF+RBV was given. Her had a RVR with undetectable viral load at week 4 that remained undetectable for the remainder of 20 weeks of SOF+RBV therapy. The strategy SOF+RBV was stopped after the 24-week therapy. A SVR was observed. The viral load remained undetectable until SVR24 (Table 2).

### 3.6 Case 5

Case 5 is a 49-year-old male with hyperthyroidism before being diagnosed with HCV genotype 1b in 2013 (Table 1). Before starting our treatment, his viral load was 2,790,000 IU/mL. The strategy SOF+RBV+PEG-IFN was given. He had a RVR with undetectable viral load at week 4 that remained undetectable for the remainder of 10 weeks of SOF-based triple therapy. The strategy SOF+RBV+PEG-IFN was stopped after the 12-week therapy. A SVR was observed. The viral load remained undetectable until SVR24 (Table 2).

### 3.7 Case 6

Case 6 is a 29-year-old male diagnosed with HCV genotype 1b in 2014. He was a patient with hyperthyroidism in March 2015 (Table 1). When after the treatment I131, he became hypothyroidism. Before starting our treatment, his viral load was 14,000,000 IU/mL. The strategy SOF+RBV was given. Her had a RVR with undetectable viral load at week 4 that remained undetectable for the remainder of 20 weeks of SOF+RBV therapy. The strategy SOF+RBV was stopped after the 24-week therapy. A SVR was observed. The viral load remained undetectable until SVR24 (Table 2).

### 3.8 Case 7

Case 7 is a 59-year-old male with diabetes II. He was diagnosed with HCV genotype 2 in 2015 (Table 1). Before starting our treatment, his viral load was 99,400 IU/mL. The strategy SOF+RBV was given. Her had a RVR with undetectable viral load at week 4 that remained undetectable for the remainder of 10 weeks of SOF+RBV therapy. The strategy SOF+RBV was stopped after the 12-week therapy. A SVR was observed. The viral load remained undetectable until SVR24 (Table 2).

### 3.9 Case 8

Case 8 is a 69-year-old female with no significant medical history before being diagnosed with HCV genotype 1b in 2007 (Table 1). Before starting our treatment, her viral load was 15,000,000 IU/mL. The strategy SOF+RBV+PEG-IFN was given. He had a RVR with undetectable viral load at week 4 that remained undetectable for the remainder of 10 weeks of SOF-based triple therapy. The strategy SOF+RBV+PEG-IFN was stopped after the 12-week therapy. A SVR was observed. The viral load remained undetectable until SVR24 (Table 2).

### 3.10 Case 9

Case 9 is a 61-year-old female with hypertension and cirrhosis. She was diagnosed with HCV genotype 1b in 2014 (Table 1). This patient received no treatment previously. Before our treatment, her viral load was 2,990,000 IU/mL. The strategy SOF+RBV+ PEG-IFN was given for 12 weeks. She had a RVR with undetectable viral load at week 4 that remained undetectable for the rest of 12 weeks of therapy. But the virus relapsed in SVR4 and was still uncontrolled in SVR12 (Table 2). The treatment failed. The patient quitted our research and turned to another therapy.

### 3.11 The expression of cytokines and chemokines

**Figure 1:**
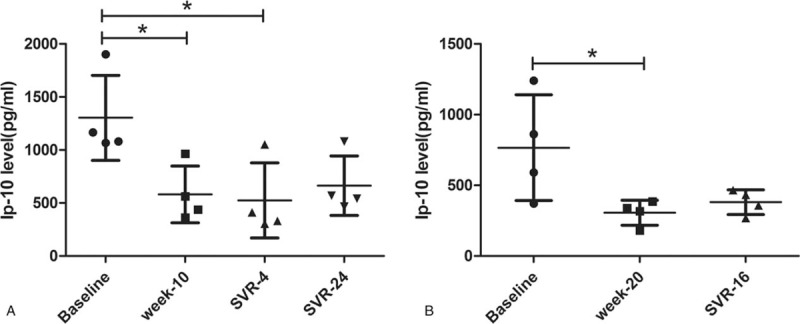
The change of IP-10 during the treatment. (A) Patients with chronic hepatitis C receiving sofosbuvir and ribavirin-based treatment with interferon; (B) Patients with chronic hepatitis C receiving sofosbuvir and ribavirin-based treatment without interferon. IP-10 = interferon-inducible protein-10, SVR = sustained virological response. ^∗^*P* < .05.

**Figure 2:**
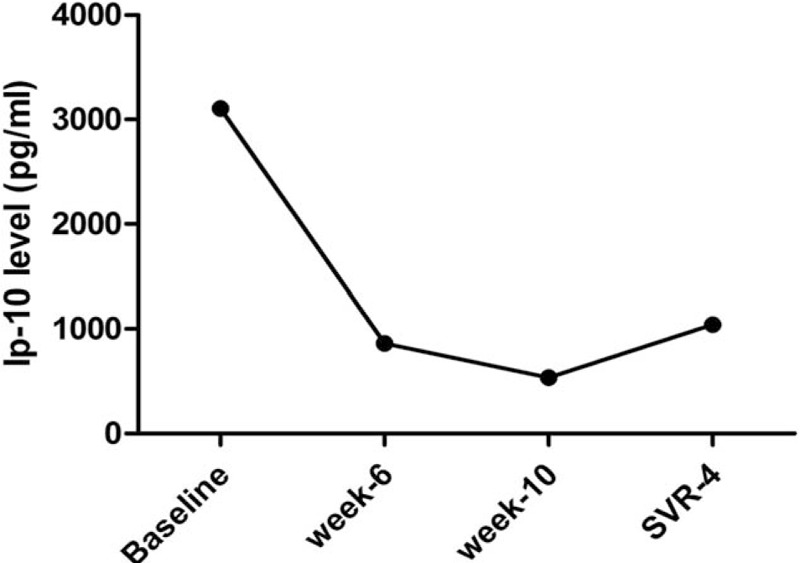
The change of IP-10 during the treatment. The patient with cirrhosis failed, as the virus reappeared during SVR4. IP-10 = interferon-inducible protein-10, SVR = sustained virological response.

The cytokines and chemokines were recorded during treatment and after treatment. A total of 38 cytokines and chemokines were tested. Eight patients were cured. The expressions of 37 cytokines and chemokines [including EGF, FGF-2, Eotaxin, TGF-α, G-CSF, Flt-3L, GM-CSF, Fractalkine, IFNα-2, IFNγ, GRO, IL-10, MCP-3, IL-12 (p40), MDC, IL-12 (p70), IL-13, IL-15, sCD40L, IL-17A, IL-1RA, IL-1α, IL-9, IL-1β, IL-2, IL-3, IL-4, IL-5, IL-6, IL-7, IL-8, MIP-1α, MIP-1β, TNF α, TNFβ, VEGF] were of no significant change after the treatment. The interferon-inducible protein-10 significantly decreased in patients with chronic hepatitis C receiving SOF and RBV-based treatment with or without interferon (Fig. 1). However, the patient with cirrhosis failed, as the virus reappeared during SVR4. At the same time, the IP-10 increased as the relapse of the HCV virus (Fig. 2).

## 4 Discussion

Until now, polyethylene glycol interferon and RBV is the mainstream treatment strategy for HCV in China.^[14]^ But there are still quite a number of patients who cannot be cured or tolerate the treatment plan. DAA was reported to be more effective and tolerable and more widely used in many nations. SOF was one of the potential DAA. In our study, we tried the SOF and RBV-based treatment with or without interferon to treat patients with chronic hepatitis C in eastern China. Nine patients participated in our research. Eight patients were cured without any severe side effects. They all had RVRs with undetectable viral load at week 4. The viral load of each patient was monitored during the treatment. They all had SVRs. No sign of recurrence was observed until SVR24. However, the patient with cirrhosis failed, as the virus reappeared during SVR4.

It is well known that cytokines and chemokines play a significant role in modulating immune response and defense against viral infection. So far, only a limited number of studies have been conducted to investigate the cytokines and chemokines in chronic HCV infections.^[15–17]^ The expression of cytokines and chemokines in patients treated with polyethylene glycol interferon and RBV has been reported. However, the expression of serum cytokines and chemokines in response to SOF and RBV-based treatment in China remains unclear.

In this study, a total of 38 cytokines and chemokines were tested during and after the treatment. Interestingly, the IP-10 significantly decreased after the treatment whether the patient was treated with or without interferon. The other 37 cytokines and chemokines showed no remarkable differences in serum concentrations after the treatment. Interferon-inducible protein-10, also known as C-X-C motif chemokine 10 (CXCL10), is a proinflammatory chemokine, which belongs to the CXC chemokine family.^[18,19]^ The IP-10 is encoded by the *CXCL10* gene, which is located on human chromosome 4.^[20]^ The IP-10 elicits its effects (chemoattraction for monocytes/macrophages, T cells, NK cells, and dendritic cells, promotion of T cell adhesion to endothelial cells) by binding to the cell surface chemokine (C-X-C motif) receptor 3 (CXCR3). IP-10 played an important role in T helper (Th) 1 type inflammatory disorders, including autoimmune, neoplastic, and infectious diseases.^[21–23]^ In recent decades, IP-10 has been studied extensively in infectious diseases such as human T-lymphotropic virus type I, dengue virus, HBV, and HCV.^[24–28]^ Most researches have shown that IP-10 has uniquely prognostic utility as a marker of treatment outcome in HBV and HCV.^[24–26,29–32]^

However, the patient with cirrhosis failed as the virus reappeared during SVR4. Meanwhile, the IP-10 dramatically increased. Treating patients with liver cirrhosis had been unsatisfactory with various degrees of failure for many years. SVR rate was low using PEG-IFN+RBV dual therapy. Conventional SOF and RBV-based treatment also failed in treating patients with cirrhosis. The mechanism was difficult to elucidate. It is possible that cirrhosis prevents even perfusion of the liver with antiviral drugs, creating pockets that have low drug concentrations where HCV can persist.^[33,34]^

Our study has limitations. First, we have a small number of patients in this study. Moreover, we monitored the change of the cytokines and chemokines during the whole process of treatment, but we lack comparable data from normal people.

In summary, SOF and RBV-based treatment were effective in treating chronic hepatitis C patients without cirrhosis. The IP-10 has the potential to be the biomarker for the prognostic of HCV. Of course, further studies with more patients and control group are desired to confirm our data.

## Author contributions

**Data curation:** Xiaowei Xu, Xiaoxin Wu, Hangping Yao, Lanjuan Li.

**Formal analysis:** Xiaoxin Wu, Hangping Yao, Lanjuan Li.

**Funding acquisition:** Xiaowei Xu.

**Investigation:** Xiaowei Xu, Xiaoxin Wu, Huilin Ou, Junwei Su, Haiying Yu, Lanjuan Li.

**Methodology:** Xiaowei Xu, Hangping Yao, Lanjuan Li.

**Project administration:** Xiaowei Xu, Lanjuan Li.

**Resources:** Xiaowei Xu, Xiaoxin Wu, Hangping Yao, Lanjuan Li.

**Supervision:** Xiaowei Xu, Lanjuan Li.

**Validation:** Lanjuan Li.

**Writing – original draft:** Xiaowei Xu, Xiaoxin Wu, Keda Chen, Dazhi Chen, Huilin Ou, Hangping Yao, Lanjuan Li.

**Writing – review & editing:** Xiaowei Xu, Xiaoxin Wu, Keda Chen, Dazhi Chen, Huilin Ou, Hangping Yao, Lanjuan Li.
